# Content validity and ePRO usability of the BPI-sf and “worst pain” item with pleural and peritoneal mesothelioma

**DOI:** 10.1186/s41687-018-0039-4

**Published:** 2018-03-27

**Authors:** Heather L. Gelhorn, Sonya Eremenco, Anne M. Skalicky, Zaneta Balantac, Tricia Cimms, Katarina Halling, Chris Sexton

**Affiliations:** 1 Evidera – Evidence, Value & Access by PPD, 7101 Wisconsin Ave, Suite 1400, Bethesda, MD 20814 USA; 2 AstraZeneca, Gaithersburg, MD USA; 3 AstraZeneca, Molndal, Sweden

**Keywords:** Qualitative, Mesothelioma, Symptoms, Pain, HRQL, Patient reported outcome, Brief pain inventory

## Abstract

**Background:**

The Brief Pain Inventory-short form (BPI-sf) is widely used in self-reported pain assessment, incorporates pain numeric rating scales (NRS) and is commonly utilized in electronic format in clinical trials, however, there is no published information about its usability as an electronic patient-reported outcome (ePRO) measure. The objective of this qualitative study was threefold: 1) to better understand pain experiences among patients with pleural or peritoneal mesothelioma; 2) to assess the interpretability of the instructions, item stem, recall period, and response option of the “worst pain” item of the BPI-sf; and 3) to examine the usability of the TrialMax Touch™ (CRF Health, Inc., Plymouth Meeting, PA) screen-based handheld device and the electronic format of the BPI-sf in a sub-sample of pleural mesothelioma patients.

**Methods:**

A cross-sectional qualitative study was conducted among participants with pleural and peritoneal mesothelioma recruited from 4 clinical sites in the US. Semi-structured telephone or in-person interviews were conducted consisting of concept elicitation, cognitive interviewing of the 11-item BPI-sf, and in-person interview evaluation of ePRO assessment usability in pleural mesothelioma patients.

**Results:**

Twenty-one participants recruited from 4 clinical sites in the US were interviewed in-person (*n* = 9) and by telephone (*n* = 12); 71% male; mean age 68.7 ± 13.6 years. Pleural and peritoneal patients described pain as ranging from discomfort to intense pain and reported being able to distinguish tumor pain from treatment pain. The BPI-sf “worst pain” item was relevant to, and easily understood by, study participants with pleural and peritoneal mesothelioma. The ePRO version was found to be easy to use, but readability of small font may be an issue. Participants reported minimal differences between their responses on the paper and ePRO version for all of the pain severity and pain interference items.

**Conclusions:**

Results support the relevance and ease of understanding of the “worst pain” item and provide support for its content validity in patients with pleural and peritoneal mesothelioma. Usability of the ePRO format of the BPI-sf was confirmed for use in clinical trials among patients with pleural mesothelioma.

**Electronic supplementary material:**

The online version of this article (10.1186/s41687-018-0039-4) contains supplementary material, which is available to authorized users.

## Backgound

Mesothelioma is a rare malignant tumor originating from the cells lining the surface of the coelomic cavities of the body, specifically the pleura, peritoneum, pericardium, and tunica vaginalis testis. Pleural mesothelioma is the most common anatomical site (67–75%) of presentation, followed by peritoneal (25–33%) [1]. Mesothelioma is mainly caused by occupational exposure to asbestos (e.g., mining, industry), and as a result, is most prevalent in men. Due to the long latency of the onset of disease after exposure to asbestos (20–40 years), cases are typically detected at an advanced age (median 72 years) and advanced disease stage [2, 3]. The incidence of malignant pleural mesothelioma (MPM) is leveling off in the United States, because asbestos use has decreased since the 1970s [4], however, it is increasing in other countries, such as Russia, Western Europe, China, and India [5].

Clinical practice guidelines acknowledge that mesothelioma has a high symptom burden including pain, fatigue, dyspnea, insomnia, cough, and anorexia [6]. Studies indicate that many patients with pleural mesothelioma report pain and/or dyspnea [7]. Patients typically present with shortness of breath due to pleural effusion or chest pain in a more advanced stage [6]. Patient pain symptoms in mesothelioma are frequently complex due to a combination of nociceptive, neuropathic, and inflammatory factors [8].

Few patient-reported instruments have been used in evaluating mesothelioma treatments. Some that have been used include the Lung Cancer Symptom Scale - Mesothelioma (LCSS-Meso) [9, 10], and the Brief Pain Inventory - short form (BPI-sf) [11]. The BPI-sf is a widely used, self-administered instrument developed to assess the severity of pain and the impact of pain on functioning [11]. The BPI-sf was originally derived from the BPI - long form which was developed to assess cancer-related pain; the BPI-sf is widely used and has evidence in support of its validity across a range of therapeutic conditions [12–14]. The BPI-sf includes front and back body diagrams, 4 pain severity items, 7 pain interference items, and an item for rating pain relief from analgesics. The 4 items of pain severity domain assess pain at its “worst,” “least,” “average,” and “now” (current pain) on an 11-point numeric rating scale (NRS) from 0 (No Pain) to 10 (Pain as bad as you can imagine). The BPI-sf uses a 24-h recall period and is designed to assess two domains: Pain Severity and Pain Interference. The BPI “worst pain” item is a commonly used endpoint for measuring pain and has been included in a number of drug labels in the US and Europe [15]. The evidence of the content validity of the BPI-sf and the “worst pain” item has not been established in patients with mesothelioma.

While electronic versions of the BPI-sf including interactive voice response (IVR) have been used in numerous studies, no published information is available about its usability in electronic screen-based format [16]. Usability testing, and/or equivalence testing may be required to confirm that the migration from the paper to electronic version has been faithful and the new implementation captures the same data as the original. In cases where there are significant changes in format when an instrument is adapted from paper to electronic format, equivalence testing might be required [17, 18]. However, as in the case of this study, if the change in format between the paper version and the ePRO version is considered to be minor only cognitive interviews and usability testing are typically conducted. This study was conducted to support the content validity of the BPI “worst pain” item for use in clinical trials of mesothelioma that may be seeking a label claim for pain improvement.

## Methods

### Study design and objectives

This cross-sectional qualitative study included concept elicitation as well as cognitive interviewing components to achieve the following three objectives: 1) to better understand pain experiences among patients with pleural or peritoneal mesothelioma; 2) to assess the interpretability of the instructions, item stem, recall period, and response option of the “worst pain” item of the BPI-sf; and 3) to examine the usability of the TrialMax Touch™ (CRF Health, Inc., Plymouth Meeting, PA) electronic format of the BPI-sf in a sub-sample of pleural mesothelioma patients (ePRO screen shots included as Additional file 1). The study was approved by local Institutional Review Boards at each clinical study site.

### Recruitment procedures

Participants were recruited from 4 US clinical sites and interviewed in-person or by telephone (for study participants who had peritoneal mesothelioma or were unable to come to the clinic). Participants included those who had: clinician confirmed diagnosis of mesothelioma, previous or current chemotherapy treatment, Eastern Cooperative Oncology Group (ECOG) performance status of 0 or 1, and were 18 years of age or older. It was not a requirement that study participants be symptomatic to participate in the study. All participants provided informed consent prior to participating in the interview and were remunerated a nominal amount for their time.

### Interview procedures

The interviews were conducted by qualitative interviewers (AS, ZB) with ten years of combined experience in conducting qualitative interviews. The interviews were scheduled to last 90 min. If time was constrained due to patient illness or time limitation due to treatment appointment or patient time constraints, the concept elicitation portion and the cognitive interview review of a separate questionnaire and the BPI-sf “worst” pain, “average” pain items, and the usability of the handheld device were priority questions.

#### In-person interviews

The in-person interviews with pleural mesothelioma participants included an evaluation of the usability of the electronic version of the BPI-sf. During the concept elicitation portion of the interview, participants were asked open-ended questions designed to elicit spontaneous descriptions of their mesothelioma symptoms. Next, the study interviewers (ZB, AS) reviewed the instructions for completing the paper version of the BPI-sf before instructing the participant to complete the questionnaire.

Participants completed a brief training on the TrialMax™ handheld device. During the training interviewers provided any needed additional explanation as to how to tap the screen to make a selection, and how to save the data. Once participants felt comfortable with the instructions for operating the electronic device, participants were asked to complete the screen-based format of the BPI-sf. Following the completion of the electronic version of the BPI-sf, the interviewer commenced the cognitive interview to evaluate participants’ comprehension and interpretation of the BPI-sf “worst pain” item (item 3) and “average pain” (item 5) and the appropriateness and ease of use of the response scales and recall periods in relation to their experience with mesothelioma. During the cognitive interview portion of the interview, pleural participants were asked to describe differences in pain concepts, such as “worst pain,” “average pain,” and “general pain.”

In the final stage of the cognitive interview, participants were asked questions related to the experience of answering questions on the screen-based handheld device, as well as additional questions focusing on the participants’ feedback on the comparison of the paper and electronic versions of the BPI-sf.

#### Telephone interviews

The telephone interviews included concept elicitation and cognitive interview of the paper version of the BPI-sf, but did not include an ePRO component. Participants were mailed a packet with study materials relevant for review during the interview. During the concept elicitation portion of the interview, participants were asked open-ended questions designed to elicit spontaneous descriptions of their mesothelioma symptoms.

Following the concept elicitation, the study interviewers reviewed the instructions for completing the paper-version of the BPI-sf before instructing the participant to complete the questionnaire. Following the completion of the BPI-sf, the interviewer commenced the cognitive interview evaluation of the participant’s comprehension and interpretation of the BPI-sf “worst pain” item (item 3) and “average pain” (item 5) and the appropriateness and ease of use of the response scales and recall periods in relation to their experience with mesothelioma. During the cognitive interview portion of the interview, participants were asked to describe differences in pain concepts, such as “worst pain,” “average pain,” and “general pain.”

### Data analysis

The project team developed a coding dictionary structured on the interview guide and based on the themes and concepts that emerged during the concept elicitation and cognitive interview discussions. One of the study team members (ZB) was responsible for supervising another coder. The coder was trained on the coding dictionary and then both ZB and the coder coded the same transcript and reviewed and reconciled their differences to improve standardized use of codes. The study project manager (AS) refereed discrepancies in coding to reconcile final coding decisions. A content analysis approach was used to analyze qualitative data from the interviews, using ATLAS.ti version 7.1.8 qualitative data analysis software [19]. Concept elicitation data were analyzed separately for participants with pleural and peritoneal mesothelioma.

Participant feedback from the evaluation of the screen-based handheld device was evaluated to determine its usability and the similarities and differences in responses between the paper and electronic formats of the BPI-sf. Descriptive statistics were used to characterize the study sample with respect to demographic and clinical characteristics using SAS version 9.1 (SAS Institute, Cary, NC).

## Results

### Demographic and clinical characteristics

A total of 21 participants recruited from four clinical sites, representing both large and small medical centers in Florida (*n* = 1), New York (n = 1) and Texas (*n* = 2), participated in this cross-sectional qualitative study, which included interviews conducted in-person (*n* = 9) and by telephone (*n* = 12) between September 2013 and June 2014. The sample included 17 participants with pleural mesothelioma, three with peritoneal mesothelioma, and one with both pleural and peritoneal mesothelioma.

Study participants were 68.7 ± 13.6 years of age (range 22.0 to 84.3 years), and the majority were male (71%). Most participants were White (*n* = 20, 95%), retired (*n* = 14, 67%), and living with a partner, family or friend (*n* = 17, 81%). Education level varied across the sample (Table 1). Participants had a mean of 1.2 (SD = 1.5) years since time of their mesothelioma diagnosis, and most were Stage III (25%) or Stage IV (60%) according to clinician diagnosis (Table 2). Eighty-six percent of the participants were on active treatment. All of the pleural participants (*n* = 18) reported undergoing treatment for their disease: primarily chemotherapy and/or surgery.

**Table 1:** Participant self-reported sociodemographic and clinical characteristics

Participant characteristics (self-report)	Total *N* = 21^a^	Pleural *N* = 18	Peritoneal *N* = 4
Age (years)^b^			
Mean (SD)	68.7 (13.6)	68.7 (14.4)	57.1 (24.7)
Range (Min, Max)	(22.0, 84.3)	(22.0, 84.3)	(22.0, 78.7)
Gender, n (%)			
Male	15 (71.4%)	13 (72.2%)	2 (50.0%)
Ethnicity, n (%)^b, c^			
Not Hispanic or Latino	19 (90.5%)	16 (88.9%)	4 (100.0%)
Racial background, n (%)^b^			
White	20 (95.2%)	17 (94.4%)	4 (100.0%)
Employment status, n (%)^b, d^			
Employed, full-time or part-time	2 (9.5%)	2 (11.1%)	0 (0%)
Retired	14 (66.7%)	12 (66.7%)	2 (50.0%)
Disabled/Other^e^	5 (23.8%)	4 (22.2%)	1 (25.0%)
Highest level of education, n (%)^b^			
Elementary/primary school	2 (9.5%)	1 (5.6%)	1 (25.0%)
Secondary/high school	5 (23.8%)	5 (27.8%)	0 (0%)
College or Postgraduate degree^b^	14 (66.7%)	12 (66.7%)	3 (75%)
Health rated within the past week			
Excellent/Very good/Good	15 (71.4%)	13 (72.2%)	3 (75.0%)
Fair/Poor	5 (23.8%)	4 (22.2%)	1 (25.0%)
Missing^b^	1 (4.8%)	1 (5.6%)	–

^a^One patient had pleural and peritoneal disease and is included in both disease-specific columns

^b^One participant did not complete the sociodemographic form

^c^One participant did not answer ethnicity question

^d^Not mutually exclusive

^e^One participant answered “student”

**Table 2:** Clinician-reported participant characteristics

Clinical characteristics	Overall *N* = 21^a^	Pleural *N* = 18	Peritoneal *N* = 4
Years since mesothelioma diagnosis			
Mean (SD)	1.2 (1.5)	1.1 (1.5)	1.4 (1.9)
Range (Min, Max)	(0.1, 4.8)	(0.1, 4.8)	(0.2, 4.2)
Stage of mesothelioma, n (%)^b^			
Stage I/Stage II	3 (14.3%)	3 (16.7%)	0 (0%)
Stage III/Stage IV	17 (80.9%)	14 (77.8%)	4 (100%)
RECIST criteria, n (%)^d^			
Partial response (PR)	4 (19.0%)	2 (11.1%)	2 (50.0%)
Progressive disease (PD)	4 (19.0%)	4 (22.2%)	0 (0%)
Stable disease (SD)	10 (47.6%)	9 (50.0%)	2 (50.0%)
ECOG performance status grade			
Grade 0: Fully active	8 (38.1%)	7 (38.9%)	2 (50.0%)
Grade 1: Restricted, light work only	13 (61.9%)	11 (61.1%)	2 (50.0%)
Comorbidities^c^			
Asthma	3 (14.3%)	3 (16.7%)	1 (25.0%)
Anxiety/depression	2 (9.5%)	1 (5.6%)	1 (25.0%)
Arthritis	2 (9.5%)	2 (11.1%)	0 (0%)
Other cancer^e^	10 (47.6%)	9 (50.0%)	1 (25.0%)
Diabetes	2 (9.5%)	2 (11.1%)	0 (0%)
Hypertension (high blood pressure)	12 (57.1%)	12 (66.7%)	0 (0%)
Chest pain/abdominal pain	3 (14.3%)	2 (11.1%)	1 (25.0%)
Other^f^	6 (28.6%)	5 (27.8%)	1 (25.0%)
None	1 (4.8%)	1 (5.6%)	0 (0%)
Current medications^b^			
Chemotherapy or other cancer treatment	18 (85.7%)	16 (88.9%)	3 (75.0%)
Nutritional support	9 (42.9%)	8 (44.4%)	1 (25.0%)
Pain medications/corticosteroids	11 (52.4%)	9 (50.0%)	3 (75.0%)
Other^g^	4 (19.0%)	3 (16.7%)	1 (25.0%)

^a^One patient had pleural and peritoneal disease and is included in both disease-specific columns

^b^One pleural participant with “unknown” status

^c^Not mutually exclusive

^d^Three pleural participants with “unknown” RECIST criteria

^e^As a requirement for study eligibility the participants had to be currently disease-free from other invasive malignancy and recovered from any toxicity related to previous or current anticancer therapies for other cancer

^f^For pleural participants: one participant reported having “GERD, gastroparesis, hyperlipidemia”; one participant reported having “allergies, hyperparathyroidism, GERD”; one participant reported having “chronic kidney disease”; one participant reported Congestive heart failure/Heart disease; one participant reported “COPD.” The one peritoneal participant reported having “anemia”

^g^Other = Correction of metabolic syndrome disorder (*n* = 3; 2 pleural, 1 peritoneal). Antibiotics (*n* = 1 pleural)

### Concept elicitation results

Forty-three percent (*n* = 9 of 21) of the overall sample described pain resulting from their mesothelioma, with the majority of these spontaneously reporting pain when asked about symptoms resulting from their mesothelioma. The location of pain varied, with participants mentioning the chest, lungs, and side, pain in the bones, or pain in general. Participants reported being able to differentiate the pain resulting from surgery and treatment compared to the pain resulting from mesothelioma. Words participants used to describe their experience of pain included “discomfort,” “twinge,” a “spot of pain,” “sharp,” and “intense.” When asked how often they experienced pain, several participants indicated the symptom was at least a daily occurrence, with others characterizing their pain as “constant.” Four participants reported that they experienced pain seldom or “every now and again,” with degree and duration ranging from an “instant twinge” to a constant ache. Of the participants who were asked about the impact of pain, several reported that their pain was bothersome.

*001-001: It’s just when it hits direct…it can be pretty rough. You know, maybe a …maybe a 7 [out of 10]. …the next time it might be a 4. …It’s almost like—you was in a fist fight and they drilled you in the chest…That’s almost how it feels.*

*001-002: it was only painful when I laid down and it was a mild pain but …it didn’t even go away and so that’s what got my attention that … I shouldn’t be having pain when I lay on that side.*

### Cognitive interview results: BPI-sf “worst pain” item

#### Instructions, recall period and response options

All of the participants in the overall sample reported a clear understanding of the instructions for completing the “worst pain” and “average pain” items. There were no comprehension issues related to the recall period of the past 24 h**.** All participants asked correctly applied the past 24-h recall in evaluating worst pain and average pain, many stating that the question was “easy” when asked whether the question was “easy or difficult to answer” in a 24-h time period. All of the participants had a clear understanding of rating pain using a NRS with many responding that it was “easy” to select a numerical value for their level of pain.

#### BPI-sf “worst pain” item stem

The concept of “worst pain,” as assessed by item 3 of the BPI-sf, was explored in several sections of the cognitive interview and in relation to the assessment of the ease of interpretability and usability of the electronic version of the BPI-sf. Participants were asked to describe how they would rate their pain within the past 24 h, including “general pain,” “worst pain,” as assessed by BPI-sf item 3, and “average pain,” as assessed by BPI-sf item 5. When asked to provide a “think aloud” response to how they interpreted the BPI-sf “worst pain” item as it was read to them during the interview, a majority of pleural participants and all peritoneal participants found the item easy to answer and focused on pain that was their most severe or intense in the previous 24-h period (Table 3).

*I would mark 0 if I had no pain at all. I would mark 10 if the pain was so intense that I was unable to essentially perform my life, you know, perform the tasks in my life (004-006).*

*So, what do you think about rating your “worst pain” over a 24 hour period?*

*“Oh, I would imagine you’d want to know how—how severe it could possibly be, right? You’re treating the severity, not just the fact that you have some pain. You want to know to what degree, so I get that.” (002-006)*

Only one-third of the pleural participants were able to describe how they would rate their “worst pain”, but reported that they were not currently experiencing pain due to mesothelioma at the time of the interview. Amongst the pleural study participants not experiencing pain, the pain item was initially described as “difficult to respond to and explain,” and some described how they felt the last time they felt pain:

*So, can you talk me through how you would answer a question about “worst pain”?*

*“I’d probably try to remember like the worst amount of pain that I had lately and like remember like when I was like, ow that hurts. And then be like, oh that sucked, and then I’d mark it down. And then, I’d try to like kind of compare it to like the “worst pain” ever. Like I don’t like giving very high numbers on pain stuff because I feel like a 10 should be like you’re basically dead from pain. So, [laughter], um, that being said, like I generally try to rate things on the lower scale because I don’t think I’ve ever experienced—like I feel like a 10 is something that I have not experienced yet because I feel like I’d die if I experienced a 10.” (003-004)*

*“Uh, so if you’d asked me two days ago maybe I would’ve said my pain in my stomach was a—a 2 or a 3 as a constant—a constant pain.” (002-010)*

In order to better understand how participants conceptualized their “worst pain,” a subset of the sample was asked to compare and contrast this concept with “general pain” and “average pain.” Most of those participants said these questions were assessing different concepts and that “worst pain” was distinctive from “average” or “general” pain. A number of participants indicated that they would respond similarly to the two questions because “worst pain” and “general pain” tended to be comparable for them within a 24-h period.

*“Um, on average I would say that like you just kind of think of like your everyday like day-to—like how it is generally like most of the time versus like the parts where it like hurts super bad.” (003-004)*

Participants were asked to use their own words to explain “average” pain. Of the participants who were asked, all of these participants talked about trying to mentally calculate their worst and least pain to find an average of their pain. These participants noted that the concept of “worst pain” was more important and straightforward to assess rather than “average pain” because the intensity or severity could be rated more easily.

*“…most of the time the pain is average and every once in a while, to me, I don’t know about other guys or other people, ah, sometimes I get sharp – sharp shooting pains, stabbing pains. Sometimes it lasts a minute, sometimes it lasts five minutes.” (002-005)*

*In your opinion, you see no difference between ‘average’ versus ‘worst’ or least?*

*Well there—there’s a mathematical difference, right? But—but if I say, what was your “worst pain” in the last 24 hours, and you say, it’s this, and, what was your least pain in the last 24 hours, and you say, that. And I—I—I essentially think Question 5 is a waste of space because you could calculate the average based on those two—those two answers (004-006).*

All of the pleural and peritoneal participants who were asked stated that they correctly applied the past 24 h recall in evaluating “worst pain” and were able to easily use the NRS. These same participants reported no difficulty when asked whether the question was “easy or difficult to answer” based on a 24 h recall period.

**Table 3:** Item Mapping of participant quotations to BPI-sf “Worst Pain” (Item 3): Pleural participants (*n* = 19)

Item	Supporting information	Relevant quotes
Item 3: Please rate your pain by marking an “X” in the appropriate box that best describes your pain at its worst in the last 24 h.	6 participants were able to describe how they would rate their “worst pain” on the BPI-sf item 3;	*“Uh, so if you’d asked me two days ago maybe I would’ve said my pain in my stomach was a—a 2 or a 3 as a constant—a constant pain.” (002–010)*
	9 participants described their “worst pain” in the past 24 h during the CI portion of the interview	*”.sometimes I get sharp – sharp shooting pains, stabbing pains. Sometimes it lasts a minute, sometimes it lasts five minutes. But on average, it is – it is, ah, it is as much as it could be.” (002–005)*

### Usability of the electronic version

Nine pleural participants (including 1 participant with both pleural and peritoneal mesothelioma) participated in the usability portion of the study, including six men and three women, ranging in age from 22 to 84 years of age (mean 67 ± 20 years).

More than half of the pleural participants reported previous experience with technology, like computers and cell phones, and the remaining reported no or very little experience with technology. All of the participants indicated that they had no prior experience using an ePRO or similar handheld device to complete a questionnaire. When asked to provide their overall impressions about using the screen-based handheld device, nearly all participants reported positive impressions about the handheld device, including that it was “convenient,” “handy,” “fast,” or “easy” to use, and none described any problems using the handheld device.

*“The questions were simple, direct. So, I didn’t think it was difficult at all.” (001-004)*

*What were your overall impressions about using this diary to answer questions? Uh, I like it actually. It’s handy. (003-004)*

The one participant who reported a negative impression about the electronic format stated that the font size was too small to be read easily. When asked about the ease of reading text on the handheld device, most participants reported that it was easy to do so. Three participants indicated that a larger font would improve ease of reading for BPI-sf items.

*It could probably be bigger—a little bit bigger. But I had to put my glasses on, but I mean [Laughter] at 69 you’ve got to wear glasses anyway. (004-001)*

*Would you change anything about the text that appears on the screen? If I didn’t have my glasses, the font could be a little larger. (002-002)*

All participants reported that it was” quick,”” no problem, “or “easy” to move through and navigate the electronic format using the screen-based handheld device. Participants reported being able to easily change their answer when necessary. When participants were asked if they experienced any difficulty entering the answer that they wanted for any of the questions on the BPI-sf, all but one of the participants reported no difficulties. The one participant reporting difficulties stated that he did not understand how to answer the body diagram question on the BPI-sf and would have liked to have had more explanation of how to complete the ePRO version.

*And how was it moving from screen-to-screen?*

*Easy, just—just touch ‘Next’. [laughter] As long as I can read N-E-X-T I can go to the next one. [laughter] And back is--goes back to the--back to—back to the previous one. I mean, that’s not hard. (002-012)*

When prompted about what might improve the electronic format, suggestions from participants included having a bigger screen, having a brighter screen, having a stylus, and improving the screen sensitivity in response to finger taps.

#### Electronic format evaluation

After discussing their overall impressions of the screen-based handheld device, participants were asked to provide feedback about the experience of completing the paper-based and electronic formats of the BPI-sf. The majority of participants selected the same response on both the paper and electronic formats for each of the BPI-sf pain severity and impact questions (Table 4). One participant noted that for item 2, which assesses the location of pain via a body diagram, his ePRO answer was different from the paper format. This the participant selected multiple locations on the paper format of the body diagram to indicate areas of pain, but it was not clear to him how to select more than one location on the electronic format. Training on how to select multiple locations of pain is recommended in order to ensure this item is completed correctly on the handheld device.

**Table 4:** Comparison of Responses on BPI-sf Electronic vs. Paper Formats: Pleural Participants (*n* = 9)

BPI-sf item	Total respondents *N* = 9 (100%)	Question not asked^a^ N (%)	Number of participants with same response on paper and electronic N (%)	Number of participants with different response between paper and electronic N (%)
Item 1: Pain today	9 (100%)	2 (18%)	7 (64%)	0 (0%)
Item 2: Diagram	9 (100%)	2 (18%)	6 (54%)	1 (9%)
Item 3: Worst pain	9 (100%)	3 (27%)	6 (54%)	0 (0%)
Item 4: Least pain	9 (100%)	3 (27%)	6 (54%)	0 (0%)
Item 5: Average pain	9 (100%)	3 (27%)	6 (54%)	0 (0%)
Item 6: Pain right now	9 (100%)	3 (27%)	6 (54%)	0 (0%)
Item 7: Medications for pain	9 (100%)	3 (27%)	6 (54%)	0 (0%)
Item 8: Pain relief	9 (100%)	2 (18%)	7 (64%)	0 (0%)
Item 9a: General activity	9 (100%)	3 (27%)	6 (54%)	0 (0%)
Item 9b: Mood	9 (100%)	2 (18%)	7 (64%)	0 (0%)
Item 9c: Walking ability	9 (100%)	3 (27%)	6 (54%)	0 (0%)
Item 9d: Normal work	9 (100%)	3 (27%)	6 (54%)	0 (0%)
Item 9e: Relationships	9 (100%)	3 (27%)	6 (54%)	0 (0%)
Item 9f: Sleep	9 (100%)	3 (27%)	6 (54%)	0 (0%)
Item 9g: Enjoyment of life	9 (100%)	3 (27%)	6 (54%)	0 (0%)

^a^Not all questions were debriefed due to interview time limit constraints

There were no other differences noted by participants in appearance and usability of the formats that may have affected how they thought about a given question or selected an answer on the BPI-sf, although participants equally expressed a preference of one format over another.

*I think the electronic is more to the point with its questions. It’s just, you know, boom. It’s just there. Where [the paper version] has got a little bit more to read or to look at where that one’s just more simplified. (001-004)*

## Discussion

This study aimed to explore the pain experience in pleural and peritoneal mesothelioma and the interpretation of the concepts captured in the BPI-sf such as “worst pain.” The BPI-sf is one of the most commonly used instruments for pain assessment, however, no previous evidence had been collected directly from patients with mesothelioma on how they experience their pain.

The concept elicitation results suggest that the study participants experience discomfort to intense pain and can distinguish mesothelioma pain from treatment pain. Study participants readily distinguished between the “average” vs. “worst” pain concepts of the BPI-sf. In particular, support was found for the concept of “worst pain,” which patients consistently interpreted using words that reflected the item’s intended meaning and may be the more reliable assessment of pain severity. Furthermore, both participants with pleural and peritoneal mesothelioma found the “worst pain” item of the BPI-sf easy to interpret and respond to using a 24-h recall period. The authors could not identify any other study examining equivalence of paper and electronic version of “worst pain” NRS [20, 21].

An additional objective of the study was to assess the usability of the electronic version of the BPI-sf which has not previously been assessed among patients with MPM and to our knowledge, is the first qualitative study to evaluate the usability of the electronic format of the BPI-sf on a screen-based handheld device. The study results are consistent with prior qualitative studies supporting the content validity of the BPI-sf in patients with cancer-related pain. Gater and colleagues [22] conducted concept elicitation interviews followed by cognitive interviews in a sample of patients with castration-resistant prostate cancer with bone metastases focusing on the BPI-sf and other pain measures. Similarly, in exploring how patients interpret the concepts of “worst,” “average,” and “current” pain in a sample of patients with bone metastases, Harris and colleagues [23] found that patients tended to consider their “worst pain” even when asked to rate “average pain”, further highlighting the reliability of the “worst pain” item. Shi and colleagues [24] explored whether current pain, “worst pain”, or pain recalled from the past week better represented cancer patients’ overall experience. Findings suggested that “worst pain” recalled over the past week contributed most to the Pain Interference score, and that current pain tended to capture less severe pain than “worst” or “average” pain recalled from the past week.

Qualitative work has also been conducted focusing solely on the “worst pain” item in 26 patients (mean age = 68, range = 44–81) with metastatic castrate resistant prostate cancer from a non-randomized Phase II study expansion cohort. Interviews were conducted to assess the comprehension of the item as well as to elicit input on the response options on the 0–10 response scale to determine the intra-patient pain rating consistency. The findings indicated that the item is well understood and that the response scale is consistent among patients, with “2” describing relatively mild, noticeable and not limiting pain, “5” describing pain that is moderate and limits activity, and “8” as severe pain that is more or less incapacitating [16].

Results from the current study suggest that the ePRO format is easily used by participants with minimal experience with technology, as evidenced by relatively few response differences between paper and electronic versions, when appropriate participant training is provided. These results are consistent with other electronic diary usability studies conducted with older adults which found no significant issues with ease of use of electronic formats [25, 26]. There is some indication that additional training and use of larger font size may be important considerations for electronic devices used with older adults [27].

A strength of the current study is that it included 21 mesothelioma patients from geographically diverse regions of the US and from multiple medical centers. Additionally, the level of evidence on migration from paper-based to electronic format is consistent with that recommended by the ISPOR ePRO Good Research Practices Task Force Reports [17, 18]. In the case of this study, the adaptation of the TrialMax™ from the paper-based format was considered minor and therefore only qualitative evidence gathered via cognitive interview and usability testing was determined to be necessary. As outlined in best practice documents, small changes made to the format of an instrument during adaptation to electronic format do not require equivalence testing [18, 28]. The similarities found in responses between the paper and electronic versions of the BPI-sf in this study are supported by a systematic review by Gwaltney and colleagues, which identified equivalent scores produced by computer and paper measures [29].

The results of the study should be interpreted in light of the following limitations. First, participants with ECOG of 0 (asymptomatic) or 1 (symptomatic but ambulatory, able to perform non-strenuous activity) were included to match the clinical trial sample. An investigation of the BPI-sf among study participants with more severe, active disease was not within the scope of this study, and therefore the extent to which the results of the current study are generalizable to this more severe population are unknown. In addition, some of the participants in the current study may not have had as many symptoms as would be expected in the context of a treatment study. Pain was less commonly described by pleural participants in this study sample than may be expected for this disease (44% of those with pleural mesothelioma; 50% of those with peritoneal mesothelioma). Only a small number of participants with peritoneal mesothelioma were identified and interviewed, one of whom had both pleural and peritoneal mesothelioma. More research is needed to better understand the experience of pain in patients with different forms of mesothelioma. Finally, during the cognitive interview of the BPI-sf the paper version was always presented before the electronic version which may have introduced systematic bias towards participants comprehension of the BPI-sf items or experience with the device.

Future work on BPI-sf electronic device format may consider larger font size or improved contrast or brightness of the screen to improve readability. In addition, investigators should encourage ePRO vendors to include options to shade and select multiple pain locations on electronic versions of the BPI-sf figures so as to enhance pain evaluation in future clinical trials.

## Conclusions

In summary, findings from this study provide strong support for the content validity of the BPI-sf in paper and electronic format for use in patients with pleural and peritoneal mesothelioma and add to existing support for the “worst pain” item as a key outcome measure in clinical trials with pleural mesothelioma patients.

## Additional file

Additional file 1: Screen Shots of Electronic Version of Instrument. (DOCX 295 kb)

## Abbreviations

BPI-sf: Brief Pain Inventory - short form
ECOG: Eastern Cooperative Oncology Group
ePRO: electronic patient-reported outcome
IVR: Interactive voice response
LCSS-Meso: Lung Cancer Symptom Scale - Mesothelioma
MPM: Malignant pleural mesothelioma
NRS: Numeric rating scale
