# Supplementary material for: Content validity and ePRO usability of the BPI-sf and “worst pain” item with pleural and peritoneal mesothelioma
Source: J Patient Rep Outcomes. 2018 Mar 27;2:16. doi: 10.1186/s41687-018-0039-4 (PMC5934933; doi:10.1186/s41687-018-0039-4)

Screen report – English (US)

Device: eDiary - HTC HD2

Version: 1 (FINAL)

**Form: introduction**

| 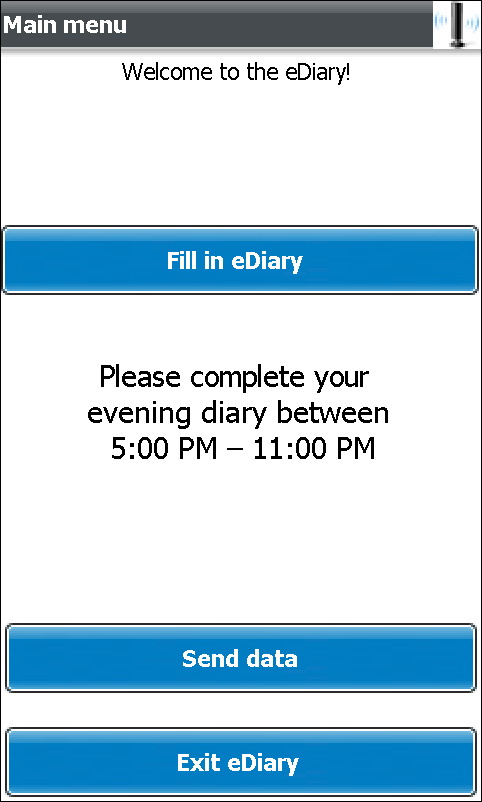Page 1 | 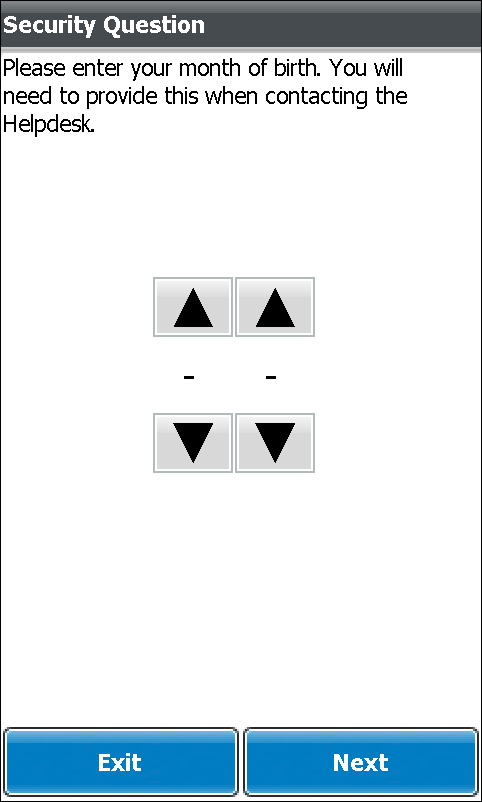 Page 2 | 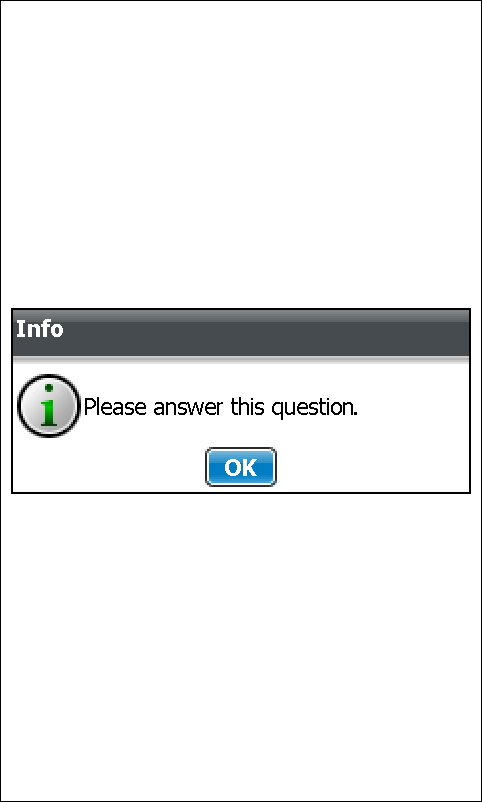  Page 3 |
| --- | --- | --- |
|  |  |  |
| **Form: LCSS-Meso** |  |  |
|  |  |  |
|  |  |  |
|  |  |  |
|  |  |  |
|  |  |  |
|  |  |  |

**Form: BPI-SF**

| 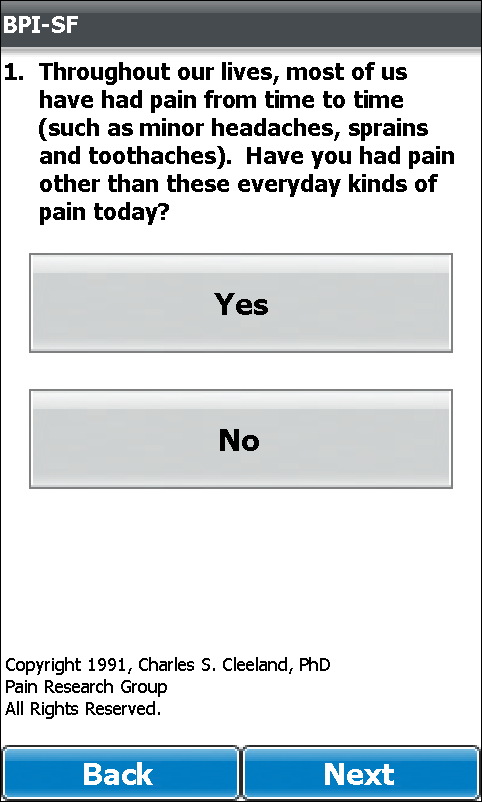  Page 1 | 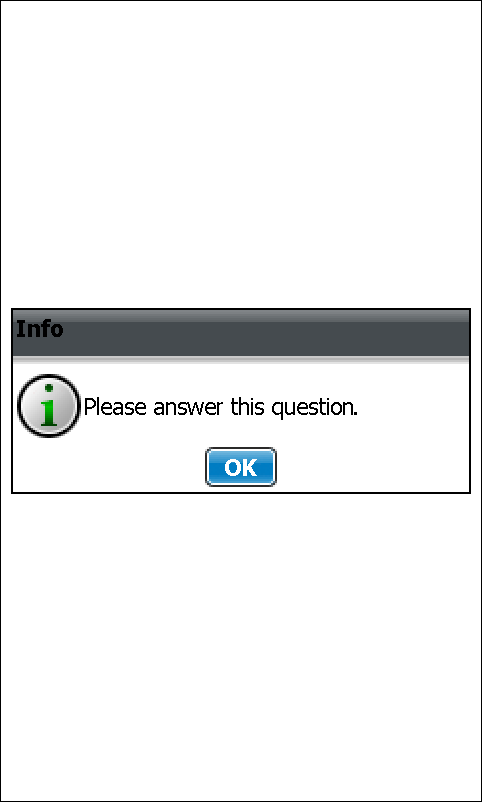 | 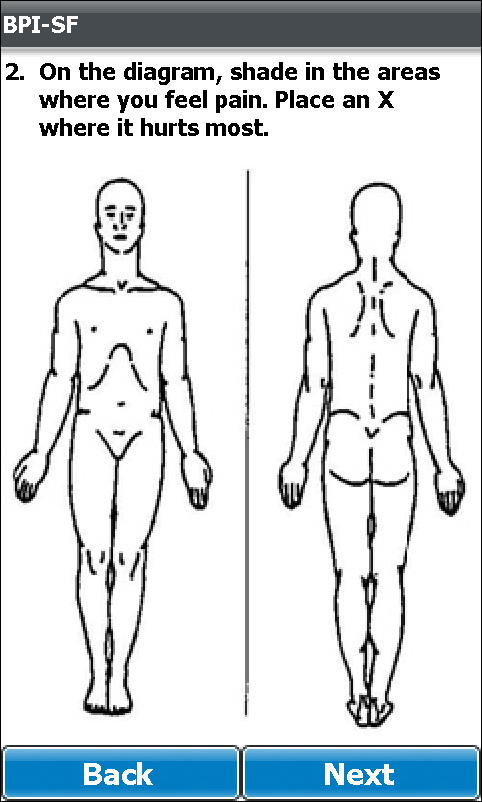  Page 2 |
| --- | --- | --- |
| 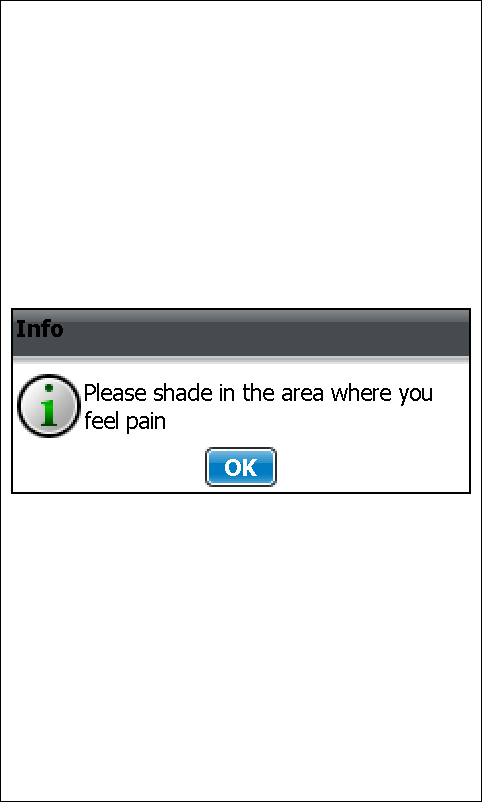 | 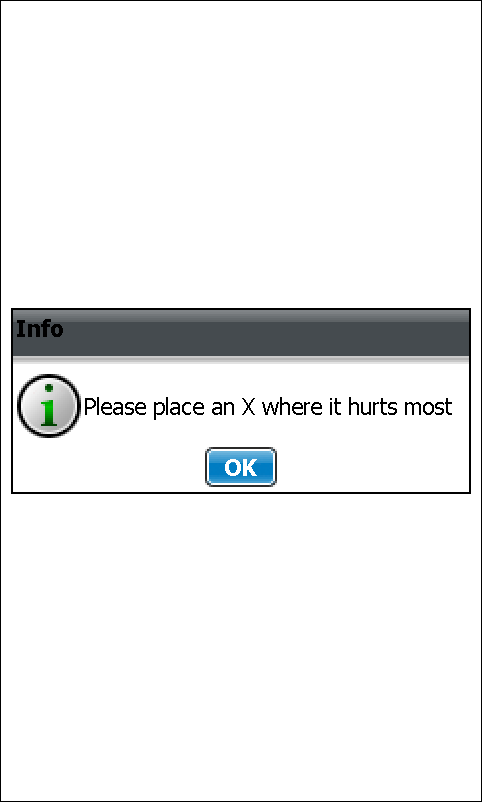 | 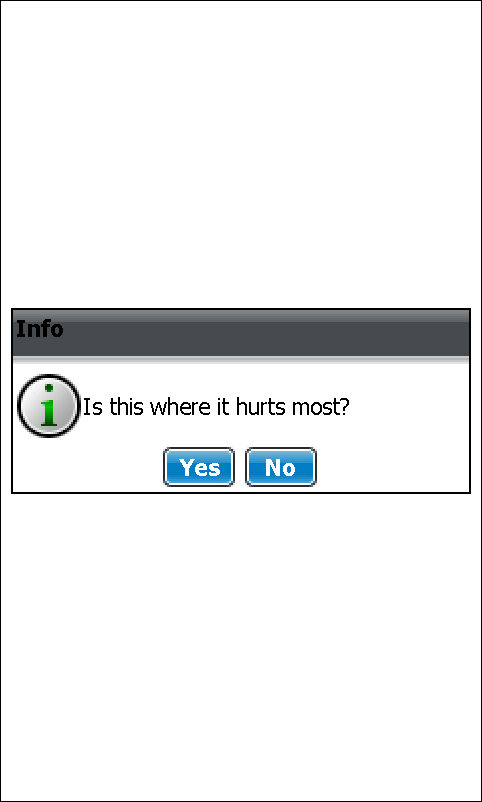 |
| 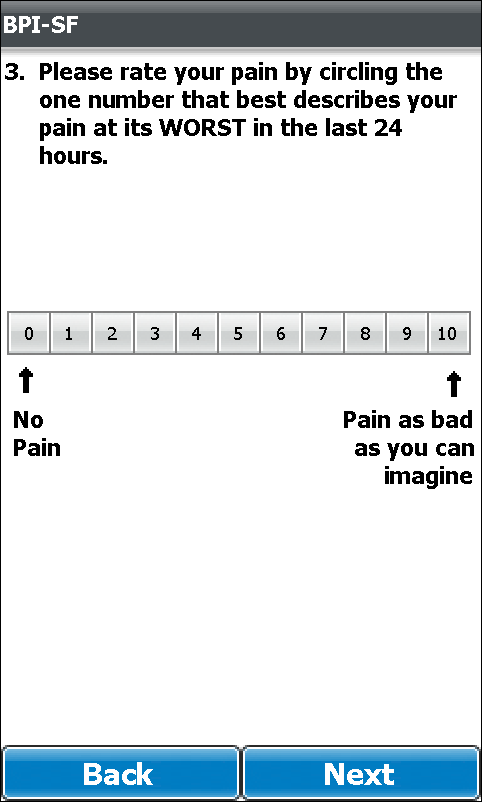  Page 3 | 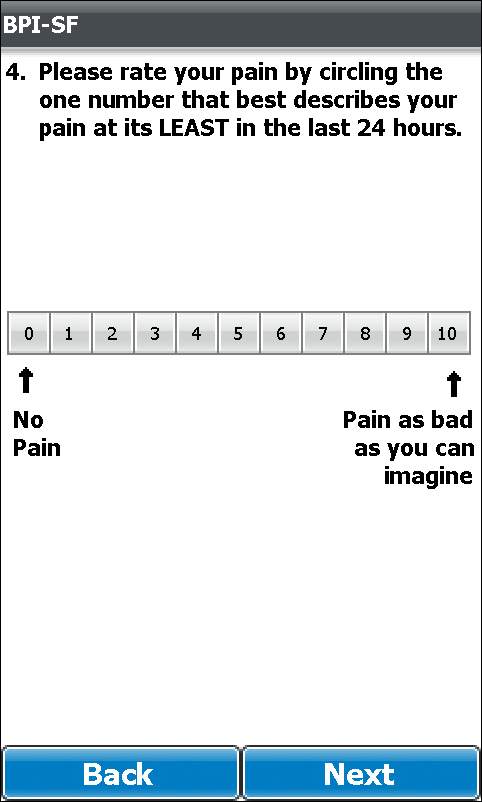  Page 4 | 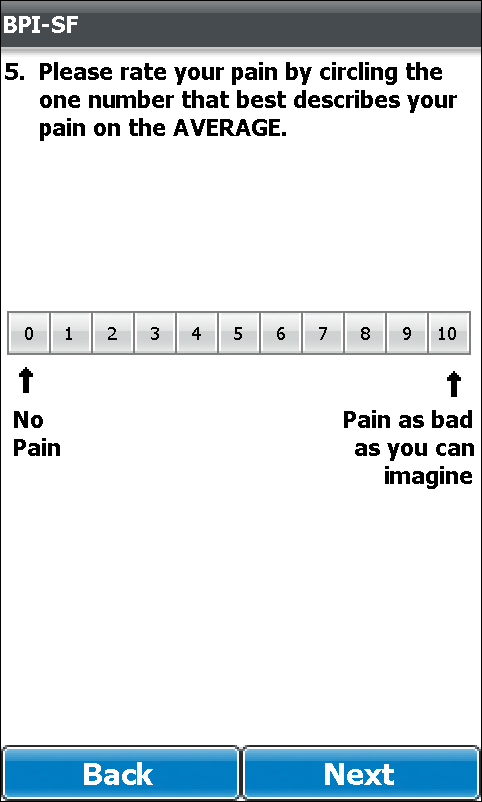  Page 5 |
| 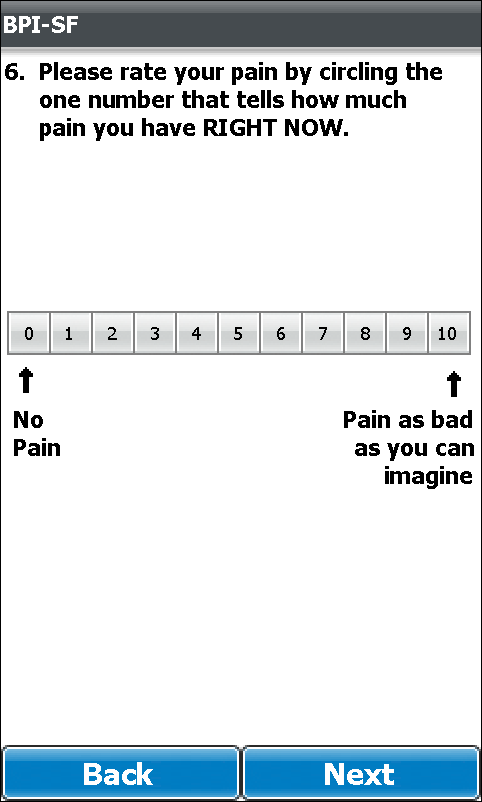  Page 6 | 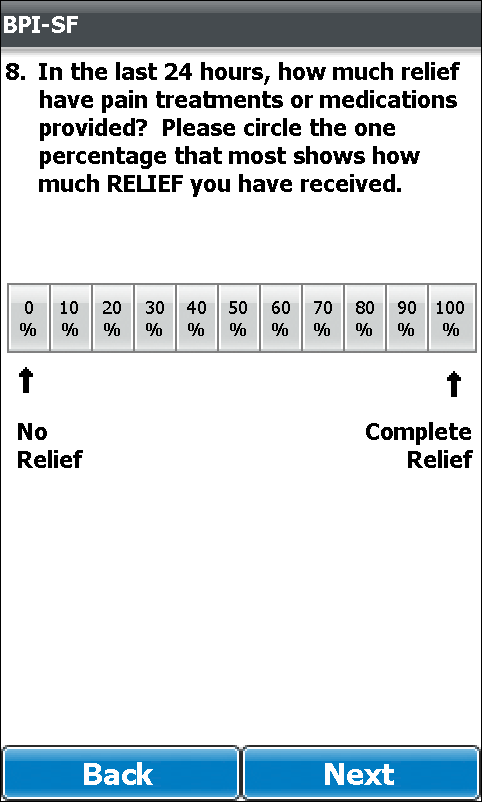  Page 7 | 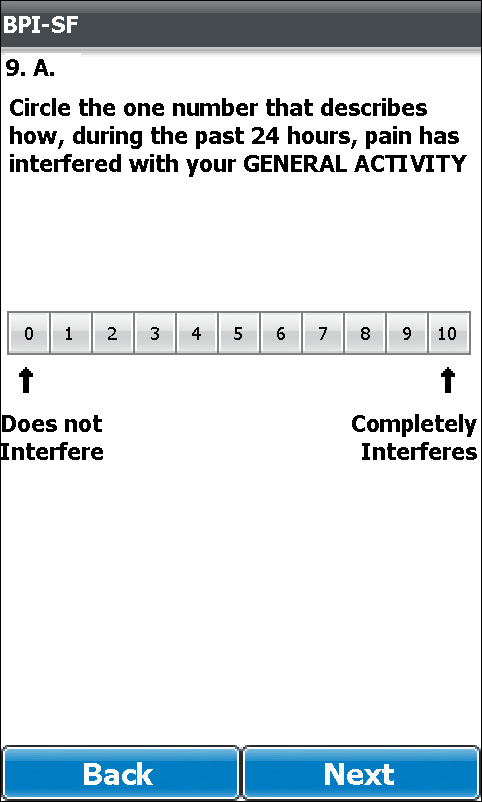  Page 8 |
| 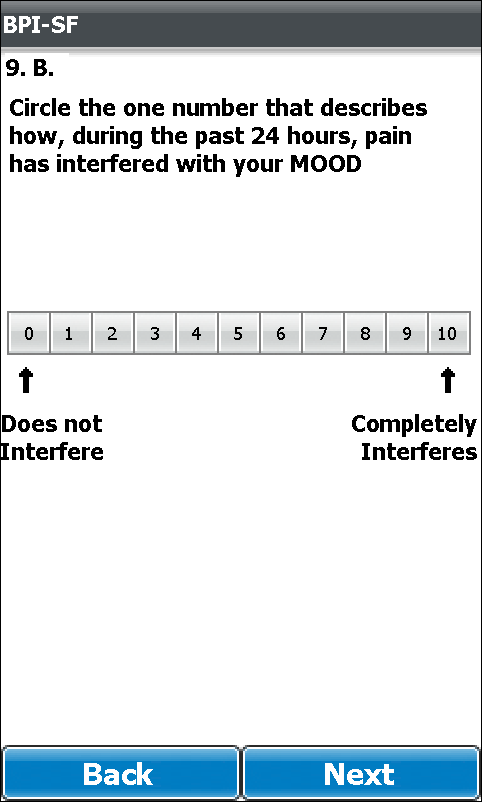  Page 9 | 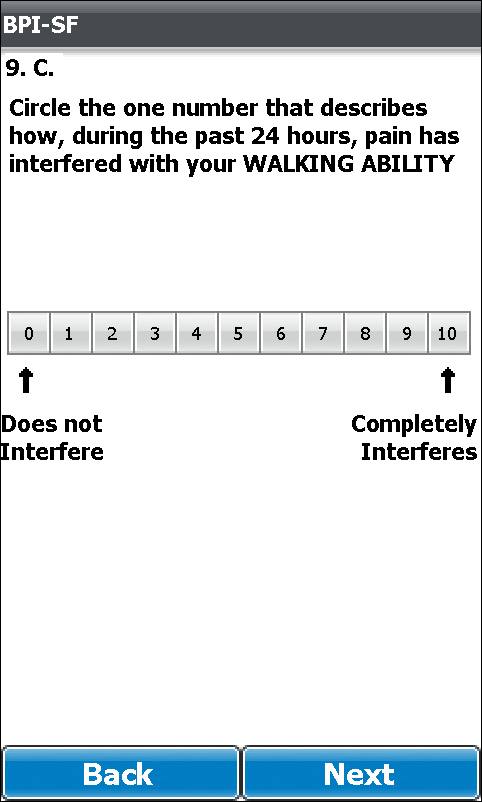  Page 10 | 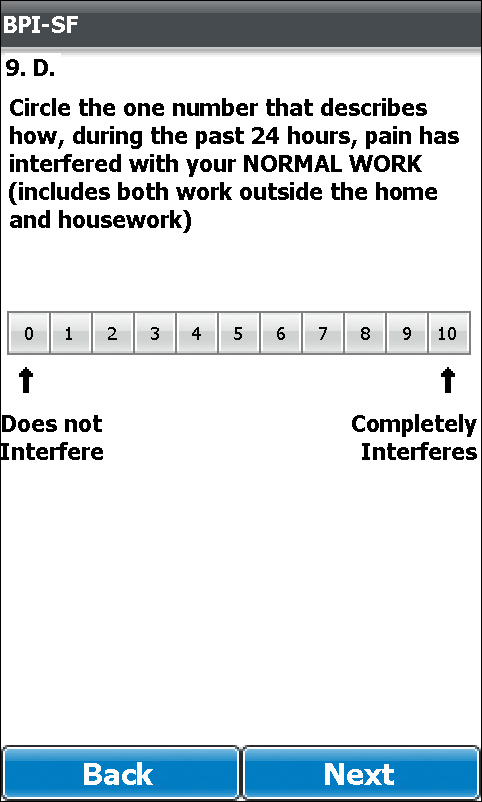  Page 11 |
| 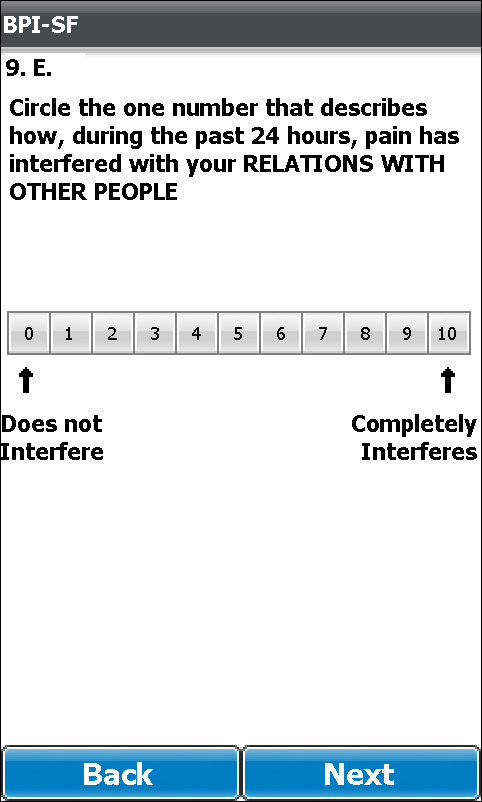  Page 12 | 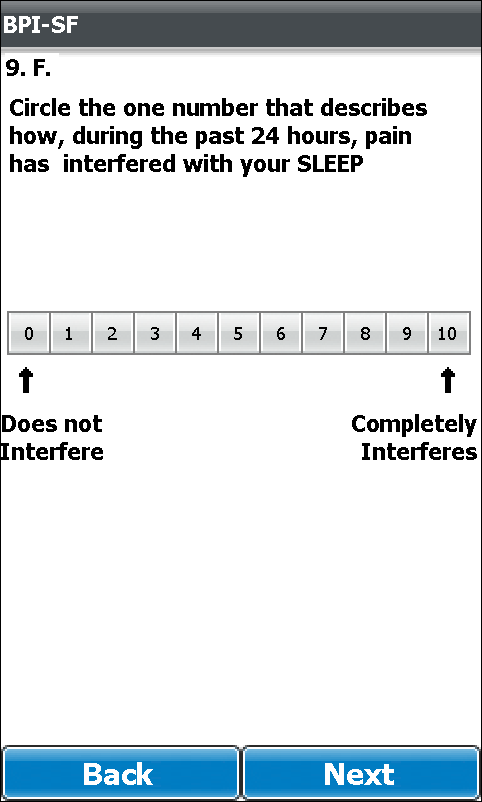  Page 13 | 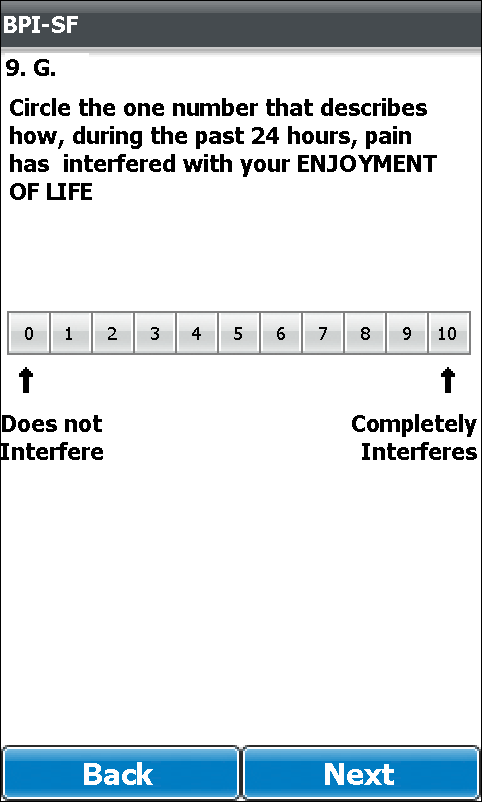  Page 14 |
| 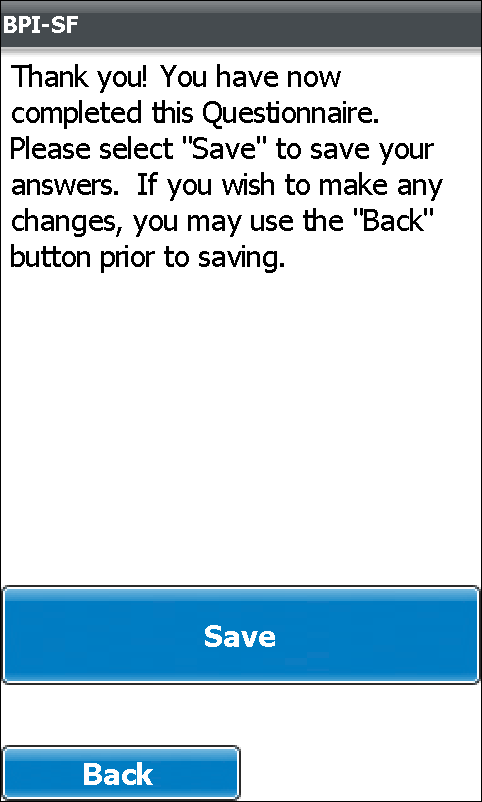  Page 15 | 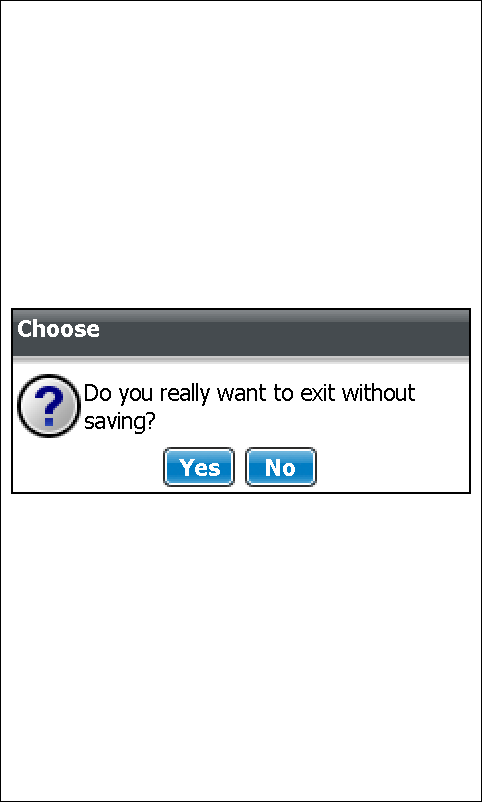 |  |
|  |  |  |

**Form: close out**

|  |  |  |
| --- | --- | --- |
| 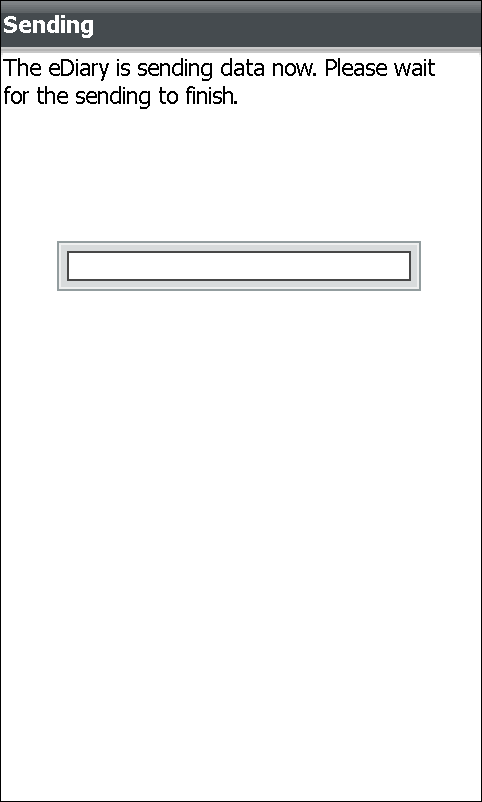  Page 12 | 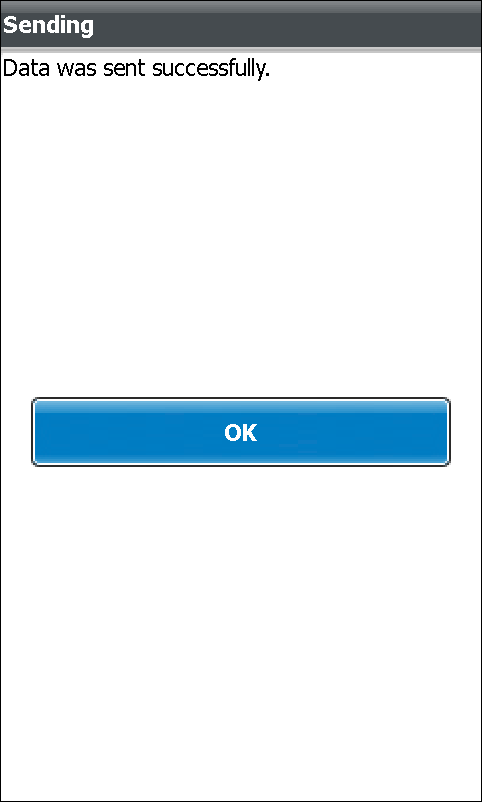 | \| 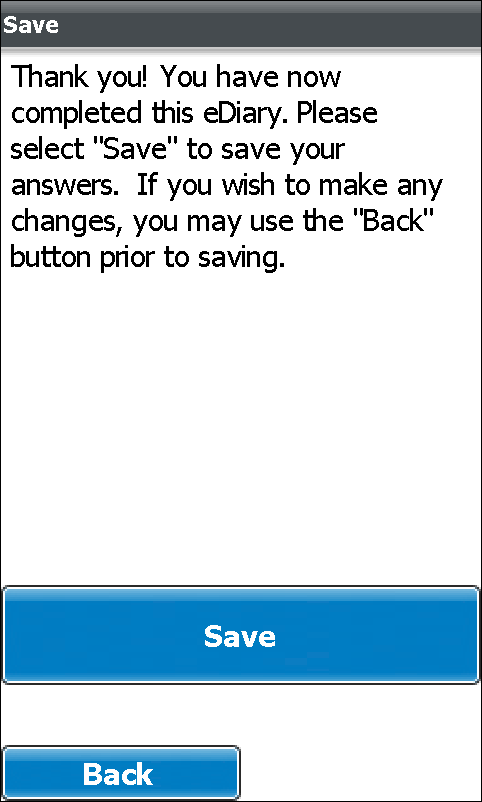  Page 13 \|  \|  \| \| --- \| --- \| --- \| |


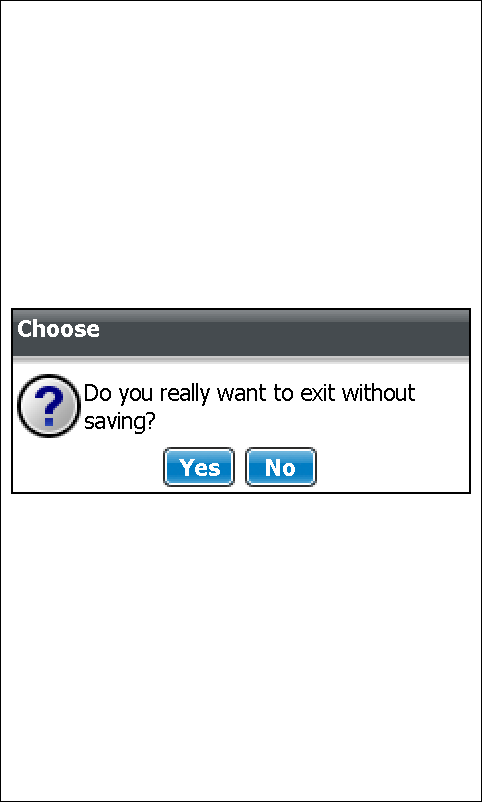

Supplement: Supplementary file 1 — Screen Shots of Electronic Version of Instrument. (DOCX 295 kb) [file 41687_2018_39_MOESM1_ESM.docx]
